# Declaration of Helsinki: ethical norm in pursuit of common global goals

**DOI:** 10.3389/fmed.2024.1360653

**Published:** 2024-04-02

**Authors:** Chieko Kurihara, Sandor Kerpel-Fronius, Sander Becker, Anthony Chan, Yasmin Nagaty, Shehla Naseem, Johanna Schenk, Kotone Matsuyama, Varvara Baroutsou

**Affiliations:** ^1^Kanagawa Dental University, Yokosuka, Japan; ^2^Ethics Working Group of International Federation of Associations of Pharmaceutical Physicians and Pharmaceutical Medicine (IFAPP), Woerden, Netherlands; ^3^Department of Pharmacology and Pharmacotherapy, Semmelweis University, Budapest, Hungary; ^4^Consultants in Pharmaceutical Medicine, Dover Heights, NSW, Australia; ^5^Pfizer Healthcare Ireland, Dublin, Ireland; ^6^The Middle East Association of Pharmaceutical Medicine Professionals, Cairo, Egypt; ^7^Academic and Research College of Family Medicine, Karachi, Pakistan; ^8^PPH plus GmbH & Co. KG, Hochheim am Main, Germany; ^9^Department of Health Policy and Management, Nippon Medical School, Tokyo, Japan

**Keywords:** Declaration of Helsinki, data-driven research, placebo, post-trial access, stakeholder involvement, health for all

## Abstract

The World Medical Association’s Declaration of Helsinki is in the process of being revised. The following amendments are recommended to be incorporated in pursuit of the common goal of promoting health for all. 1. Data-driven research that facilitates broad informed consent and dynamic consent, assuring participant’s rights, and the sharing of individual participant data (IPD) and research results to promote open science and generate social value. 2. Risk minimisation in a placebo-controlled study and post-trial access to the best-proven interventions for all who need them. 3. A future-oriented research framework for co-creation with all the relevant stakeholders.

## 1 Introduction

The Declaration of Helsinki (DoH) of the World Medical Association (WMA) (1), first adopted in 1964, is the world’s most widely recognised ethical principle for medical research involving humans. The WMA began the process of revising the DoH in April 2022, from the last version dated 2013. Research involving humans is a core activity in the development of medicines. For this reason, the authors have discussed the ideal function of the ethical norm of research involving humans, considering our global experience of the COVID-19 pandemic and other disasters, including war situations. The DoH is a fundamental ethical norm, not guidance for specific changing situations. However, as described below, the drastic changes in both global society and the scientific environment over the past decade have posed an acute challenge to this fundamental norm.

## 2 Ethics in data-driven research

### 2.1 The Declaration of Taipei and broad informed consent

The WMA’s first declaration on health databases in 2002 was triggered by the nationwide genome biobank planned in Iceland around the time of the completion of the human genome draught sequence. It was revised in 2016 as the Declaration of Taipei (DoT) (2) on health databases and biobanks. However, the latest version of the DoH does not mention the DoT. Recently, the secondary use of real-world data (RWD) from clinical practise or data generated from research has been widely accepted, particularly with the rapid development of artificial intelligence. RWD are also used as external controls (3) to compare new intervention with natural history of disease rather than conducting placebo-controlled trials. Furthermore, the COVID-19 pandemic has raised an acute demand for data-driven public policy, not limited to health policy. Meanwhile, the European Union’s General Data Protection Regulation (4) and the proposed regulations of the European Health Data Space (5) seek to increase the potential for secondary use of personal data within a strengthened governance framework whilst guaranteeing individuals’ rights to control their data and increasing data portability. In such an environment, clarification of the link between the DoH and the DoT is essential (6, 7). The DoT is not limited to the protection of privacy and data security. It sets out a governance framework including the management of incidental findings, intellectual property rights, and material transfer agreements, which must be explained to the individuals who consent to the multipurpose use of their data. Such a type of consent is called “broad informed consent” in the guidelines of the Council for International Organisations of Medical Sciences (CIOMS) (8), as opposed to both orthodox informed consent for use with an explicit purpose and traditional broad blanket consent. This concept of broad informed consent can enhance the common understanding of the emerging environment of data-driven research amongst researchers, research ethics committees, research participants, and society at large.

### 2.2 Rights to know/not to know and dynamic consent

The DoH guarantees research participants the right to know “*the general outcome and results of the study*”. However, it does not guarantee research participants the “right to know or not to know” (9) both incidental findings and study target outcomes, depending on the level of scientific validity, clinical significance, and actionability. These rights are endorsed in the CIOMS guidelines (8) and incorporated into some regional guidance (10). On the other hand, the International Conference on Harmonisation’s Good Clinical Practise (ICH-GCP) (11–13) does not assure these rights. Therefore, in pharmacogenomics studies and other clinical trials to develop therapeutics with biomarkers, including those for infectious diseases, the ethical responsibility of the physician investigator to inform study participants of clinically significant results generated by biomarkers without marketing authorization may become difficult. For this reason, these rights should be aligned and recognised within authoritative international norms such as the DoH.

Research participants should also be guaranteed the right to be informed about the secondary use of their data and the possible consequences, as well as the right to withdraw their consent to further use of their data. Consent that guarantees such rights is called “dynamic consent” (14, 15). Mechanisms to ensure dynamic consent can be achieved through an improved data management structure, as it requires informing individuals about secondary use projects, using advanced information technology tools, and terminating the use of data from individuals who have withdrawn amongst a large number of data subsets. Management and handling of broad informed consent and dynamic consent should be described both in the protocols and informed consent forms and evaluated by research ethics committees. The approach for informing participants on using their data for secondary studies should be carefully described.

### 2.3 Individual participant data sharing and result registration for open science with social value

Registration of “individual participant data (IPD) sharing plan” (16) and “results” of a clinical trial in a public database (17) have become regulatory requirements in various countries (18) but are not explicitly mentioned in the DoH. In the United States (19) and the European Union (20), open science has been promoted by ensuring public access to peer-reviewed papers and their supporting data from publicly funded research. As data from research involving humans is recognised as a public good (21), it should be reaffirmed as an ethical obligation of researchers to disclose not only the research results but also the IPD sharing plan in public databases.

There is also an urgent need to ensure the quality of data-driven research whilst guaranteeing the right of individuals to control their own data. The CIOMS guidelines define “social value” not just “scientific value” as the ethical justification for research. The mechanism to ensure scientific integrity, including responsible data management, to generate social value, using personal data with/without explicit consent but gaining social consensus, must be established. For this reason, “social value” should be defined in the DoH as a requirement for any type of research.

## 3 Placebo control and post-trial access

### 3.1 Risk minimization in placebo control

Controversy over the DoH article on the placebo-controlled trial has spanned approximately 30 years and, unfortunately, has led to unsuccessful attempts to develop pragmatic guidelines. The DoH should restore the original pursuit of ideals as the ethical duty of physicians (22–24). In 1975, it was clearly stated that the interests of research participants must prevail over the interests of science and that every patient in research should be assured of the best-proven method (25). Thus, since 1975, it has been recommended that a new intervention be compared with a proven intervention. This is based on the Declaration of Geneva (26) and the International Code of Medical Ethics (27), which clarify the duty of physicians to patients. The justification for a comparative study has been recognised as “clinical equipoise” (28) or “uncertainty” (29) between the arms being compared. This ethical norm is not “deceptive” (30–32), because it is independent of the statistical methodology used, with the intention to reject the null hypothesis of a significant difference in efficacy. The DoH’s current notion of the risk threshold, “no increase in serious or irreversible harm” in the control group, is not consistent with the policy of risk minimisation that applies to all types of research, not just comparative trials.

### 3.2 Post-trial access for all

The debate on placebo control raised a norm in the 2000 version of the DoH regarding the right of trial participants to post-trial access to interventions proven to be effective. This was to avoid injustice and exploitation of the host community of a placebo trial in low- and middle-income countries (LMICs), which may not have access to a high-priced intervention that has been shown to be effective (33, 34). In subsequent revisions, it also came to be a pragmatic guideline requiring to describe a plan for post-trial access in the study protocol and informed consent form. Approximately two decades later, our unprecedented experience with the COVID-19 pandemic led to a significant shift in practise. Governments, in cooperation with companies and other stakeholders, made maximum efforts to provide vaccines proven to be effective to those who needed them around the world. The post-trial access, achieved for COVID-19 vaccines due to the solidarity and collaboration amongst stakeholders in the global community represents progress, although not a universal success. Bilateral negotiations between companies and governments in high-income countries have neutralised the ideal of equitable vaccine distribution set out by COVAX (35). Some initiatives of technology transfer and capacity development have been sought in the pursuit of common global goals (36, 37), to overcome inequity and injustice in the right to health (38). “Post-trial access for all” should not be seen as idealism. It should be clearly recognised as the international principle and ethical obligation of the government, sponsors, researchers, and relevant stakeholders, including health technology assessment bodies, in support of the global availability of the best-proven interventions and access for all those who need them.

### 3.3 Obligation of care

Other unprecedented situations of clinical trials in war/conflict, as well as natural disasters, highlighted the needs of patients seeking access to investigational intervention (39, 40). Sponsors, investigators, and regulators (41–43), undertook joint efforts to continue or start investigational treatment for patients with acute needs, and developed procedures for adherence to GCP under disruptive circumstances, including the cases of emigrations. Access is not only the issue of post-trial but also the issue of patients’ right to health and the obligation of care of the physician (8). Research is now an integral part of the health system and people’s lives (44). This is the same in both normal and emergency settings. We should also assure hospitals and other points of care, as well as patients, that they must be protected under neutrality principles (45) during conflicts. We have to find agreed-upon solutions for acute conflicting values in the name of “justice.” Post-trial access must be rephrased and recognised as a human rights norm, superseding any inequity, injustice, or inhumanity.

## 4 Future-oriented framework for co-creation

### 4.1 Interdisciplinary study team and patient public involvement

The DoH has been the model for more than half a century with its paternalistic nature to clarify an individual physician’s obligation to an individual patient (46). Meanwhile, authors participate in the Ethics Working Group (EWG) of the International Federation of Associations of Pharmaceutical Physicians and Pharmaceutical Medicine (IFAPP). IFAPP was founded in 1975 as a Federation of National Member Associations, composed mainly of physicians engaged in the development of medicines. In 2018, taking into account the multidisciplinary collaboration of different expertise needed, IFAPP updated its Code of Ethics to a new Ethics Framework (47) that clarifies the shared responsibility of different experts involved in all aspects of medicine lifecycle management.

In the current decade, greater involvement of patients, the public, and bioethicists has been needed, taking into account not only normal but also catastrophic situations. For this reason, we strongly endorse the norm of shared responsibility amongst interdisciplinary teams, along with the promotion of patient and public involvement (PPI) (14). PPI activities should be evaluated to ensure that they adequately protect and do not unduly influence patients or the public. It is worth noting that our comments for the revision of the DoH have been constructed through extensive communication with and learning from patient and public positioning groups or individuals. For example, in Japan, patients and citizens, who have been well emancipated through a systematic educational programme (48), have expressed their own opinions on the DoH (15) with the aspiration for social value in research, ensuring the dignity and rights of research participants.

### 4.2 Diversity in study participants, and in ethical review

In addition, we need principles of inclusiveness that apply to vulnerable populations, providing them equitable access to promising investigational interventions within a robust framework of risk and benefit assessment and avoiding “therapeutic misconception” (misunderstanding of research as therapy). The diversity of participants in clinical trials is also essential to ensuring the generalisability of trial results (48, 49). Inclusiveness and diversity are also needed in the membership of research ethics committees to assess the values and perspectives of these various study participants and emerging new scientific methodologies, such as decentralised clinical trials, adaptive designs, and pragmatic trials, which may sometimes include cluster randomisation (50). Research Ethics Committee membership must be appointed in a fair and transparent manner.

The study evaluation system in these dynamic situations, including disaster settings, must incorporate strengthened situational adaptive nature and procedures. Innovative ethical review systems should be developed, such as generic protocol review during normal times and rapid expedited review in times of disaster; as well as reviewing the clinical use of unproven interventions with, e.g., Bayesian statistical methods to evaluate safety and efficacy according to the collection of case data. Such studies would require appropriate data quality and integrity oversight.

### 4.3 Research not limited to medical, as co-creation with study participants

Finally, to achieve the protection of research participants and research integrity in such an evolving environment, we need to recognise study participants as partners in co-creation (51). Various types of research, not only medical and health-related but also social, behavioural, educational, engineering, environmental, and space development, have become subject to ethical principles. This suggests the need to change the key terminologies from “medical research involving human subjects” to “research involving humans (or human participants)”.

## 5 Conclusion

The DoH, a living document (52), has continued to uphold its nature as a code of ethics for a physician conducting research, with the utmost respect for the dignity and human rights of an individual research participant. It reminds us that the physician–patient relationship, whilst it exists within the context of a dynamic community and global society, continues to be paramount. The altruism of participants could be fulfilled by knowing that the results of the research contribute to people with common sufferings worldwide. The ethical principles of research involving humans must be in pursuit of the common goal of promoting the health and wellbeing of every member of our global community. For this reason, we recommend the following to be incorporated in the next revision of the DoH:

- Data-driven research that facilitates broad informed consent, dynamic consent, and data sharing for open science generating social value.
- A plan to minimise the risk for placebo-controlled studies, and post-trial access to best-proven interventions for all who need them.
- Future-oriented research framework for co-creation amongst interdisciplinary teams, patients and the public, research ethics committees, and all other relevant stakeholders.

## Author contributions

CK: Conceptualization, Data curation, Formal analysis, Investigation, Methodology, Project administration, Supervision, Writing – original draft, Writing – review & editing. SK-F: Conceptualization, Investigation, Methodology, Supervision, Writing – original draft, Writing – review & editing. SB: Writing – original draft, Writing – review & editing. AC: Writing – original draft, Writing – review & editing. YN: Writing – original draft, Writing – review & editing. SN: Writing – original draft, Writing – review & editing. JS: Writing – original draft, Writing – review & editing. KM: Project administration, Supervision, Writing – original draft, Writing – review & editing. VB: Conceptualization, Data curation, Investigation, Methodology, Project administration, Supervision, Writing – original draft, Writing – review & editing.
